# Self-organizing human cardiac microchambers mediated by geometric confinement

**DOI:** 10.1038/ncomms8413

**Published:** 2015-07-14

**Authors:** Zhen Ma, Jason Wang, Peter Loskill, Nathaniel Huebsch, Sangmo Koo, Felicia L. Svedlund, Natalie C. Marks, Ethan W. Hua, Costas P. Grigoropoulos, Bruce R. Conklin, Kevin E. Healy

**Affiliations:** 1 Department of Bioengineering, University of California, Berkeley, California 94720, USA.; 2 California Institute for Quantitative Biosciences, Berkeley, California 94720, USA.; 3 Department of Material Science and Engineering, University of California, Berkeley, California 94720, USA.; 4 Gladstone Institute of Cardiovascular Disease, San Francisco, California 94143, USA.; 5 Department of Medicine, University of California, San Francisco, California 94143, USA.; 6 Department of Cellular and Molecular Pharmacology, University of California, San Francisco, California 94143, USA.; 7 Department of Mechanical Engineering, University of California, Berkeley, California 94720, USA.

## Abstract

Tissue morphogenesis and organ formation are the consequences of biochemical and biophysical cues that lead to cellular spatial patterning in development. To model such events *in vitro*, we use PEG-patterned substrates to geometrically confine human pluripotent stem cell colonies and spatially present mechanical stress. Modulation of the WNT/β-catenin pathway promotes spatial patterning via geometric confinement of the cell condensation process during epithelial–mesenchymal transition, forcing cells at the perimeter to express an OCT4+ annulus, which is coincident with a region of higher cell density and E-cadherin expression. The biochemical and biophysical cues synergistically induce self-organizing lineage specification and creation of a beating human cardiac microchamber confined by the pattern geometry. These highly defined human cardiac microchambers can be used to study aspects of embryonic spatial patterning, early cardiac development and drug-induced developmental toxicity.

## Abstract (web summary)

Organogenesis is orchestrated by biochemical and biophysical stimuli. Here, Ma *et al*. generate a micro-patterned surface that provides mechanical cues which, when combined with biochemical signals, drive human pluripotent stem cells’ differentiation into beating cardiac microchambers resembling primitive hearts.

## Graphical abstract

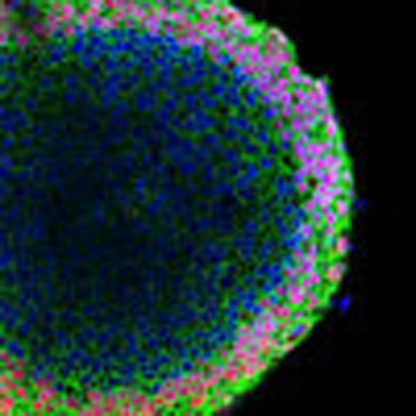

Over a century ago biophysical properties of tissue patterning were postulated as important factors in tissue development^1^. In the context of embryogenesis and morphogenesis, the developing form of the embryo and the phenotype of the organism are not only the direct consequences of transcription networks and molecular signalling^2^,^3^, but are also influenced by fundamental biomechanical processes dictated by physical laws. In heart development and congenital heart diseases, the developing tissues form in response to complex biophysical interactions of looping structures^4^, yet this is difficult to model *in vitro*. Over the past decade, there have been a number of advances that exploit material-based approaches to control *in vitro* stem cell-fate determination and lineage specification. Material control of cell differentiation has spanned manipulation of substrate stiffness^5^,^6^,^7^, spatial surface patterning^8^,^9^,^10^,^11^,^12^,^13^,^14^ and surface topography^15^. Many of these studies focused on how mesenchymal stem cells, or progenitors of differentiated cells, were able to sense mechanical forces and biophysical cues from their environment and transduced those signals through a variety of pathways to specifically regulate cell fate and lineage specification. For example, micropatterning mesenchymal stem cells has been used to study the biochemical and biophysical cues during tissue morphogenesis, where the pattern’s boundary spatially provided passive mechanical stimuli to regulate actomyosin contractions and the RhoA/ROCK pathway during differentiation^9^. These studies have raised questions concerning how biophysical cues act in concert with well-defined biochemical signalling pathways to spatially determine cell fate during tissue morphogenesis.

Emergent properties residing in the stem cell population are thought to direct organ development by responding to biochemical and biophysical cues. Controlling the differentiation of pluripotent stem cells through cell patterning has also been used as an *in vitro* model for systematically studying mammalian embryogenesis and embryonic spatial patterning^10^,^12^,^13^,^16^. Previously, we observed that embryoid bodies generated from human-induced pluripotent stem cells (hiPSCs) spontaneously organized into cardiac tissue with blood-filled vessels (Supplementary Movie 1), but they lacked the biophysical controls to produce microchambers in a controlled, reproducible manner that could be used for experimental analysis. To address this issue, we developed a cell-patterning method to present long-lasting biophysical cues to hiPSCs during the entire cardiac differentiation process (∼15 days). This allowed us to make a direct association between spatial cell-fate specification because of the confinement conditions and the formation of a beating three-dimensional (3D) cardiac microchamber, which can be used to model aspects of early developing heart.

Here we report that cell condensation is associated with biased cell migration and differential cell proliferation at the perimeter of individual patterned hiPSC colonies, and such events directed the spatial differentiation of hiPSC during mesoderm induction. This early lineage specification resulted in the formation of 3D cardiac microchambers with cardiomyocytes in the centre and myofibroblasts on the perimeter of individual microchambers. As a clinical example, we demonstrated the use of these cardiac microchambers for developmental drug-screening to improve the safety of medications administered during pregnancy.

## Results

### Patterned PEG-based substrates spatially organize hiPSCs

**Figure 1:**
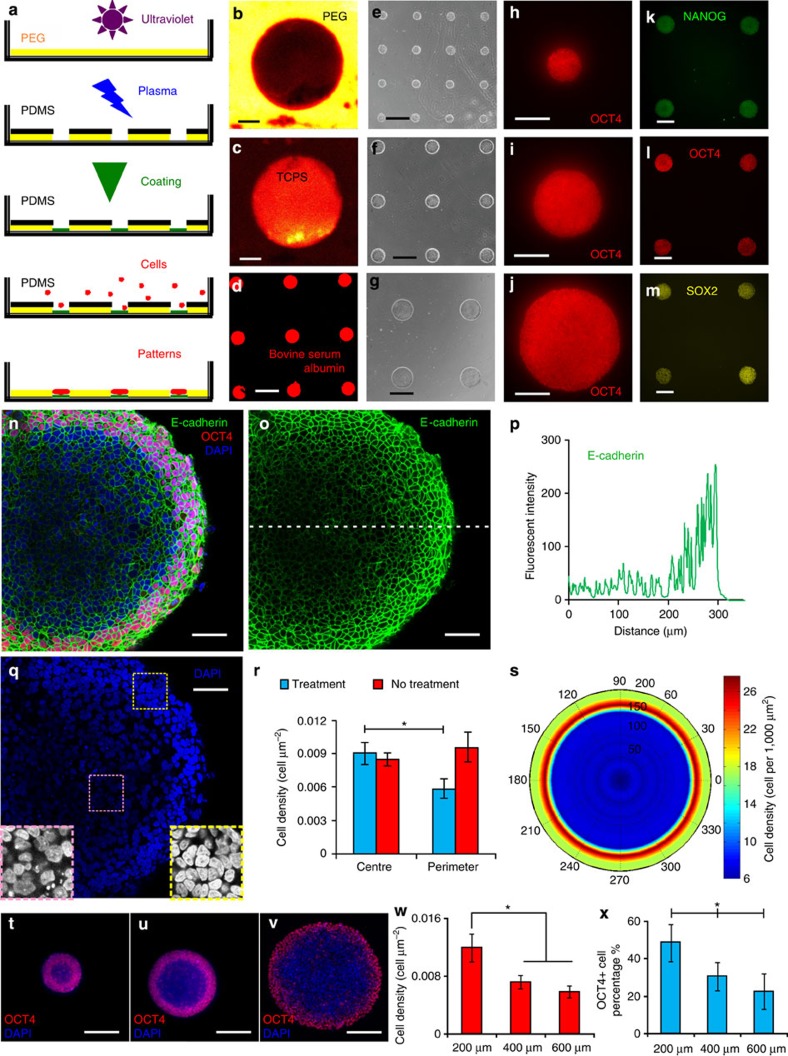
Spatial differentiation of patterned hiPSCs. (**a**) Schematic of the fabrication process for PEG-patterned substrates confirmed by ToF-SIMS imaging to show the (**b**,**c**) PEG-related peak and TCPS-related peak. Scale bar, 50 μm . (**d**) Only the etched region can adsorb bovine serum albumin. Scale bar, 600 μm. hiPSCs were patterned as (**e**–**g**) arrays of circles with different diameters (scale bar, 600 μm), and (**h**–**m**) they maintained their pluripotency (scale bar, 200 μm). After CHIR treatment, the confocal images showed (**n**) cells retained OCT4 expression as an annulus adjacent to the pattern perimeter with (**o**,**p**) higher E-cadherin expression and (**q**,**r**) higher cell density. Scale bar, 50 μm. Data represent as the means with error bars s.d. with *n*=20 individual patterns. Statistical comparison was made between the centre and perimeter using two-sided Student’s *t*-test. *P<0.05. (**s**) The heatmap of cell density for the 400-μm circle showed that the highest cell density occurred at the interface between OCT4+ and OCT4− cells. (**t**–**v**) The OCT4+ annulus was observed on all patterns. Scale bar, 200 μm. (**w**,**x**) Patterns with 200-μm diameters had highest cell density and highest OCT4+ cells compared with 400- and 600-μm diameter patterns. Data represent as the means with error bars s.d. with *n*=20 for each pattern size. Statistical comparison was made among different pattern sizes using one-way analysis of variance (ANOVA) with *post hoc* Tukey tests. *P<0.05.

To pattern hiPSCs for an extended period of culture, we grafted a poly(ethylene glycol) (PEG)-based non-fouling polymer film to standard tissue culture polystyrene (TCPS), and then patterned the substrate by masking it with a microfabricated polydimethylsiloxane (PDMS) stencil and subsequent exposure to an oxygen plasma to etch the PEG film from the TCPS substrate (Fig. 1a). We confirmed that the PEG layer was grafted on and etched from the TCPS using X-ray photoelectron spectroscopy (XPS, Supplementary Fig. 1); moreover, time-of-flight secondary ion mass spectrometry (ToF-SIMS) showed distinct PEG-related peaks outside the etched area and polystyrene-related peaks within the etched area (Fig. 1b,c). The PEG layer repelled protein adsorption (Fig. 1d), and a thin edge of the PEG layer outside the etched area retained cells within the patterned domain and prevented outgrowth (Supplementary Fig. 2). By coating the PEG-patterned surface with Matrigel, we were able to create patterns of human embryonic stem cells (hESCs, H9, Supplementary Fig. 3) and hiPSCs (WTC, Fig. 1e–g). The patterned cells were maintained in chemically defined media (that is, mTeSR) and homogeneously expressed NANOG, OCT4 and SOX2 across all the patterns (diameters of 200, 400 and 600 μm; Fig.1h–m, Supplementary Fig. 3a).

### Cell condensation induces formation of an OCT4 annulus

We employed these spatially resolved interfaces to help understand the coordination between biophysical and biochemical cues during early heart development. We followed a protocol that uses small molecules to modulate the WNT/β-catenin pathway to differentiate patterned hiPSCs into cardiomyocytes^17^. After cells proliferated to fill the patterns (∼3 days), we used a GSK3 inhibitor CHIR99021 (CHIR) to activate the canonical WNT/β-catenin pathway for mesoderm induction. After 1 day of treatment, cells in the centre of the patterns lost OCT4 expression; however, the cells at the perimeter continued to express OCT4 (Fig. 1n; Supplementary Fig. 3b) and showed higher expression of E-cadherin compared with the centre (Fig. 1o,p), which indicated that these cells exhibited an epithelial phenotype. In contrast, N-cadherin expression of cells in the centre indicated a mesenchymal phenotype (Supplementary Fig. 4). E-cadherin has been well known for maintaining pluripotency and inhibiting differentiation of hiPSCs^18^, while the assembly and disassembly of E-cadherin responds to the mechanical environment^19^, which in turn elicits cell-signalling events^20^, modulates actin dynamics^21^ and contributes to collective cell migration^22^.

By examining the confocal images of 400-μm circular patterns, we found that the perimeter region had a higher cell density than the centre region after CHIR treatment (Fig. 1q,r). Then, by plotting cell density as a heatmap, we identified an OCT4+ annulus adjacent to the pattern perimeter coincident with the region of higher cell density, suggesting that mesenchymal condensation occurred at this epithelial–mesenchymal interface (Fig. 1s; Supplementary Fig. 4). Mesenchymal condensation occurs as mesenchymal cells aggregate before differentiation, which represents an early stage of organ formation^23^. This observation was independent of the ECM proteins used to coat the substrate (Supplementary Fig. 5). In our case, the cells undergoing epithelial–mesenchymal transition (EMT) condensed their colonies at the pattern perimeter because they were geometrically confined. We also observed that the 200-μm patterns had a higher cell density over the entire pattern (Fig. 1t–w), which correlated with more OCT4+ cells than 400- and 600-μm patterns (Fig. 1x). To verify the relationship among the size of patterned area, cell density and OCT4+ cells after CHIR treatment, we created an asymmetric donut-shaped pattern with a narrow area and a wide area. In this pattern, the narrow area had a higher cell density, more OCT4+ cells and higher E-cadherin expression, whereas cells in the wide area only retained OCT4 expression at the perimeter (Supplementary Fig. 6).

### Synergism of migration and proliferation in cell condensation

**Figure 2:**
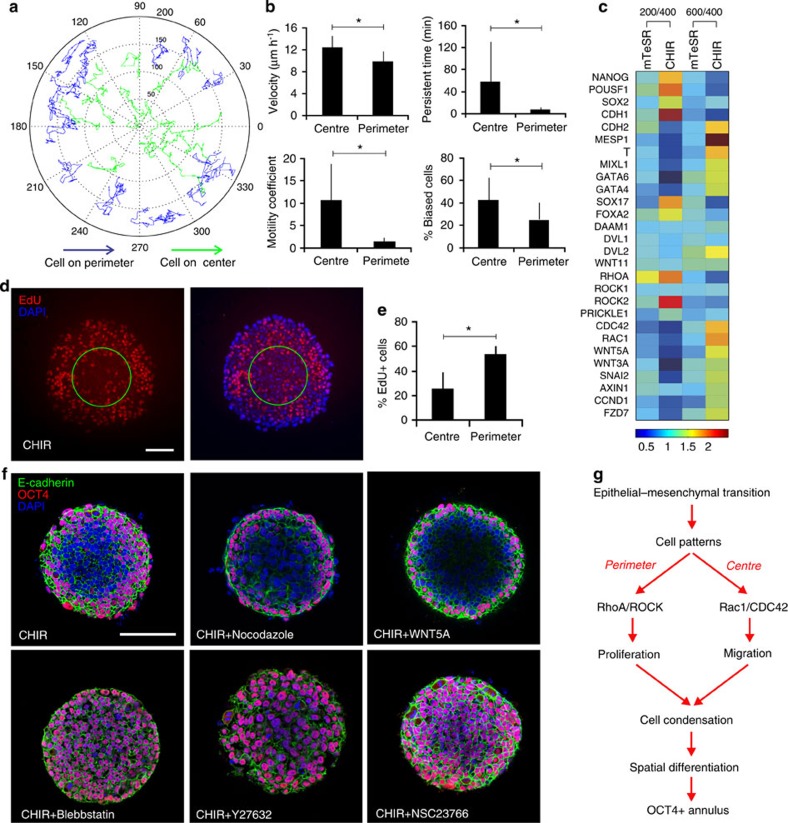
Biophysical cues directed the spatial differentiation. (**a**) Single-cell tracking showed that cells in the centre of the pattern had higher potential to migrate directionally towards the perimeter during the EMT with a higher (**b**) migratory velocity, motility coefficient, persistent time and percentage of ‘biased’ cell migration. Data represent as the means with error bars s.d. with *n*=50 for five individual patterns multiplied by 10 cells within each pattern. Statistical comparison was made between centre and perimeter using two-sided Student’s *t*-test. *P<0.05. (**c**) Gene expression for cells on the 200- and 600-μm diameter circle patterns, normalized to the 400-μm diameter patterns, before and after CHIR treatment. (**d**) EdU staining to visualize the cell proliferation on 400-μm diameter patterns. (**e**) More EdU+ cells were located near the pattern perimeter compared with the centre. Data represent as the means with error bars s.d. with *n*=20 individual patterns. Statistical comparison was made between the centre and perimeter using two-sided Student’s *t*-test. *P<0.05. (**f**) Confocal images of 200-μm patterns treated with CHIR and different compounds that modulate cell mechanotransduction. (**g**) A proposed mechanism of OCT4+ annulus formation on the patterns relies on cell condensation during the EMT, via a combination of cell migration and proliferation, to generate a high-cell-density annulus adjacent to the pattern perimeter. Scale bars, 100 μm.

One mechanistic model for mesenchymal condensation suggests that cell compaction results from directional migration of cells from the surrounding loose mesenchyme to the condensed region^24^. Therefore, we used time-lapse microscopy to monitor and analyse the *in vitro* cell condensation process during the EMT within the 400-μm patterns. We found that a population of cells in the centre of the pattern migrated radially towards the perimeter during CHIR exposure, which, over time, resulted in a higher cell density at the pattern perimeter (Supplementary Movie 2). Using a single-cell tracking software, we analysed cell migration within the patterns and classified the cells with directional migration as ‘biased’ compared with the cells with random walk, which we characterized as ‘random’ (Fig. 2a, Supplementary Fig. 7). During EMT, cells in the centre migrated faster and exhibited biased migration with concomitant higher motility and longer persistence time than did the cells at the perimeter (Fig. 2b).

Cell condensation has also been associated with smaller cell sizes and distorted cell shape^24^. Owing to the substrate confinement and biased outward cell migration, cells on the perimeter exhibited elongated nuclear shapes with higher nucleus shape indices (for example, epithelial-like apical-basal polarity), whereas the cells in the centre remained rounder (Supplementary Fig. 8a–d). This indicated that the perimeter cells were under high mechanical stress, which led to higher RhoA pathway activation on the perimeter cells compared with the centre cells (Supplementary Fig. 8e,f). Creating triangle patterns and sharp-angled patterns that had the same area as the 400-μm circle patterns, we found that cells along the edges had distorted morphologies, especially at the angled points of these patterns (Supplementary Fig. 9). Local high mechanical forces, linked to RhoA/ROCK activity of the cells, promote cell proliferation and tissue growth at the perimeter of micropatterns^25^. Therefore, we performed an 5-ethynyl-2′-deoxyuridine (EdU) incorporation study to visualize the cell proliferation of patterned hiPSCs during CHIR treatment, and found more EdU+ cells at the perimeter than in the centre (Fig. 2d,e). Therefore, cell condensation at the pattern perimeter resulted from the combination of biased cell migration of cells to the pattern perimeter and enhanced cell proliferation of cells at the perimeter (Fig. 2g).

### Biophysical cues direct cell condensation and lineage specification

To investigate the relationships between pattern sizes, cell-fate determination and cell mechanical signalling, we analysed gene expression of cells within circle patterns of three different sizes before and after CHIR treatment (Fig. 2c). We found that cells in the smaller patterns after CHIR treatment exhibited higher pluripotent markers (NANOG, POUSF1, SOX2 and CDH1), more primitive endoderm differentiation (SOX17 and FOXA2), lower canonical WNT pathway activation (WNT3A, SNAI2, AXIN1, CCND1 and FZD7), less mesoderm differentiation (T and MIXL1) and less early cardiac lineage induction (MESP1, GATA4 and GATA6) compared with the larger patterns. We found that gene expression of signalling pathways related to cell polarity (WNT5A, DAAM1 and DVL2) and mechanotransduction (RAC1, CDC42, RHOA and ROCK2) increased after CHIR treatment. We also observed increasing motility-related gene expression (RAC1 and CDC42), but decreasing elongation-related gene expression (RHOA and ROCK2) as pattern size increased.

To further investigate how mechanical signalling affected cell condensation and cell-fate determination, we tested various modulators (Supplementary Table 1)^26^ of mechanotransduction to disrupt the hiPSCs’ response to biophysical cues (Fig. 2f). We quantified cell condensation using the nucleus shape index (Supplementary Fig. 10a) and spatial cell density ratio (Supplementary Fig. 10b) for the patterns treated with CHIR and modulators. Reducing cells’ mechanical sensing by interfering the RhoA/ROCK pathway (Y-27632), myosin light-chain kinase (Blebbistatin) and Rac1/CDC42 pathway (NSC23766), we found hampered cell condensation. On the other hand, enhancing cell traction forces (Nocodazole) and cell polarity (WNT5a recombinant protein) promoted the cell condensation on the patterns. We also found that modulators promoting cell condensation produced a narrower OCT4+ annulus near the pattern perimeter, while modulators inhibiting cell condensation eliminated annulus formation (Fig. 2f, Supplementary Fig. 10c). These results indicated that the level of cell condensation directly affected the spatial differentiation of hiPSCs on the patterns.

### Formation of well-defined cardiac microchambers

**Figure 3:**
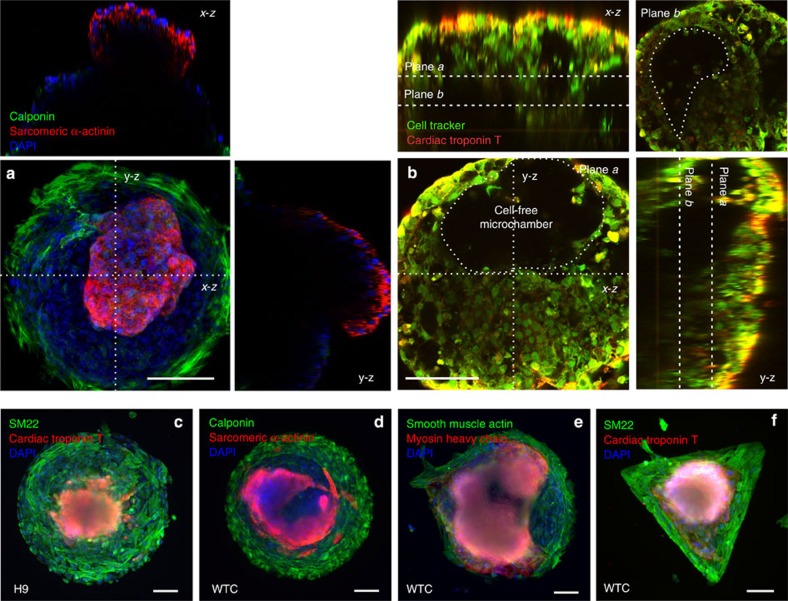
Generation of spatially organized 3D cardiac microchambers. (**a**) 3D cardiac microchamber generated from WTC hiPSCs on a 400-μm pattern, where cardiomyocytes only appeared in the centre and myofibroblasts on the perimeter. Panels above and to the right of the main panel represent the *z* axis projection images at their respective *x* and *y* cross-sections. (**b**) Two-photon microscopy image of the void inside the 3D cardiac microchamber with the cell-free chamber shown at two chamber heights (plane *a* and *b*). The *z* axis projection images at their respective *x* and *y* cross-sections are shown in panels above and to the right of the main panel. (**b**) The image through plane *a*, and the panel in the upper right shows the image through plane *b*. The cardiomyocytes in the centre were positive for (**c**) cardiac troponin T, (**d**) sarcomeric α-actinin and (**e**) myosin heavy chain, whereas the myofibroblasts on the perimeter were positive for (**c**) SM22, (**d**) calponin and (**e**) smooth muscle actin. (**f**) Spatially organized cardiac microchambers were also generated on a triangle pattern with 540-μm leg length and similar area to a 400-μm circular pattern. Scale bars, 100 μm.

To study the effect of colony size and biophysical cues on cardiac tissue formation, we continued the cardiomyocyte differentiation protocol on the patterns with different sizes. On Day 15, cardiac differentiation only succeeded on the 400- and 600-μm diameter circle patterns, and on the triangle patterns with the same surface area as the 400-μm circles (Fig. 3), but not on the 200-μm circle patterns, suggesting that cardiac tissue formation was sensitive to the size of the developing tissue rather than the micron-scale shape. In patterns that produced successful cardiac cells, we identified beating cardiomyocytes in the centre of the 3D microchambers (Supplementary Movie 3), which was confirmed using confocal microscopy, and myofibroblasts on the perimeter (Fig. 3a). To further observe the internal structure of these 3D microchambers, we used two-photon microscopy to enhance the penetration depth of the imaging and found small cell-free regions inside the microchambers (Fig. 3b, Supplementary Movie 4). Spatial cardiac differentiation was observed in H9 hESCs (Fig. 3c) and WTC hiPSCs (Fig. 3a,d–f). Cells in the centre expressed the cardiomyocyte markers cardiac troponin T (cTnT), sarcomeric α-actinin and myosin heavy chain, whereas cells on the perimeter expressed the myofibroblast markers SM22, calponin and smooth muscle actin^27^ (Fig. 3c–f). Using motion-tracking software developed in-house, we located the beating cardiomyocytes in the centre of the pattern shown in contraction heat maps and quantified their contraction behaviour (Supplementary Fig. 11, Supplementary Movies 5 and 6).

**Figure 4:**
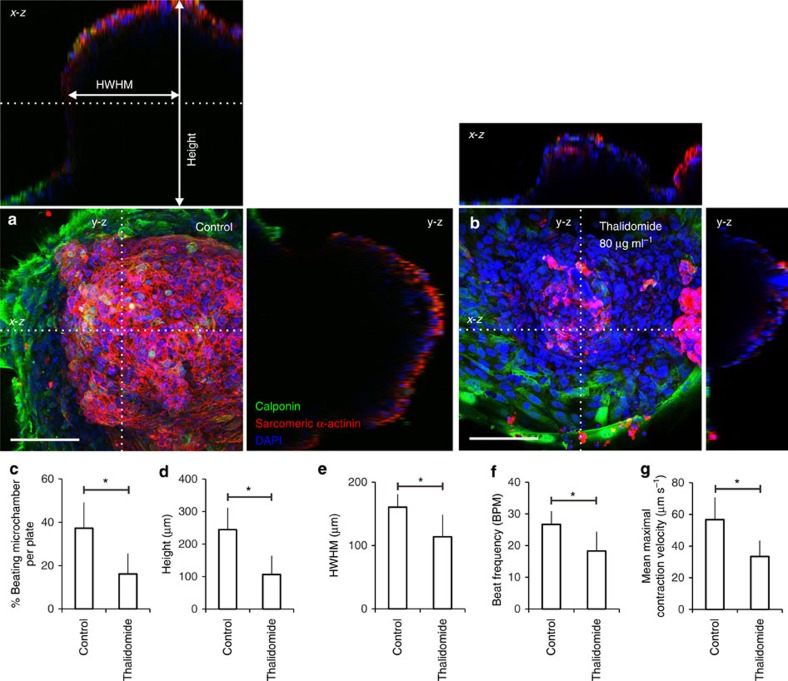
Assessment of cardiac developmental toxicity. (**a**) 3D cardiac microchamber generated from WTC hiPSCs on a 600-μm circular pattern. (**b**) Microchamber generated from WTC hiPSCs on a 600-μm circular pattern after exposure to Thalidomide during the cardiac differentiation. Panels above and to the right of the main panel represent the *z* axis projection images at their respective *x* and *y* cross-sections. (**c**) Thalidomide exposure reduced the cardiac differentiation efficiency, (**d**) lowered the microchamber height, (**e**) reduced the microchamber half width half maximum, (**f**) decreased the beat frequency and (**g**) decreased the contractility (for example, contraction velocity). Data represent as the means with error bars s.d. with *n*=6 for each experimental condition. Statistical comparison was made between the control group and drug group using two-sided Student’s *t*-test. *P<0.05. Scale bars, 100 μm.

We chose drug cardiac developmental toxicity screening to demonstrate a clinically relevant application of the cardiac microchambers. Each year, as many as 280,000 pregnant women are exposed to drugs with evidence of potential fetal risk^28^. The most commonly reported birth defects involve the heart, and the potential for generating cardiac defects is of upmost concern in determining drug safety during pregnancy. Current developmental toxicity tests with conventional two-dimensional (2D) culture and differentiation of hiPSCs^29^ are not ideal drug-screening assays because of their inability to predict the effects of drugs on tissue morphogenesis at early cardiac development stages. Therefore, we used this *in vitro* cardiac microchamber to mimic the early developing human heart to screen drugs likely to generate to cardiac birth defects. Thalidomide, with conclusive clinical outcomes of cardiac birth defects, was used to validate our model. Cases of thalidomide embryopathy have been described after maternal ingestion of as little as one 50-mg capsule. Thalidomide consumption during pregnancy has been reported to induce cardiovascular malformations ranging from ventricular and atrial septal defects, to complex conotruncal defects, and early deaths^30^. Complex, especially conotruncal defects, lesions were seen among early deaths. We found that applying this drug at 80 μg ml^−1^ during the cardiac differentiation not only reduced the differentiation efficiency, but also significantly damaged the cardiac microchambers, generating chambers with smaller size, lower contractility and beat rates compared with controls (Fig. 4).

## Discussion

These cardiac microchambers, reminiscent of early stages of cardiac development, ranged in height from 100 to 300 μm. Cardiac embryogenesis and development involve precisely regulated molecular and embryogenetic events, triggered by specific signalling molecules and mediated by tissue-specific transcriptional factors and epigenetic regulation. At the early differentiation stage with 1-day CHIR treatment, activation of the WNT/β-catenin pathway recapitulated early embryogenesis, including reduction in pluripotent markers, EMT induction, mesoderm specification (for example, expression of T and MIXL1) and spatial patterning (Supplementary Fig. 12). These observations are consistent with those for human ES cells and mouse embryos depicting emergence of the primitive streak and transition toward mesoderm^31^. Expression of NODAL, BMP and WNT signalling contributed to the onset of cardiac differentiation with increases in cardiac progenitor markers (for example, MESP1 and GATA4/6), which modelled the early cardiac lineage specification^32^,^33^. Inhibition of the WNT signalling led to the beginning of the microchamber formation. These cardiac microchambers with cell-free regions are reminiscent of early stages of cardiac development, at the stage the linear heart tube has formed and the onset of ventricular bulges from the tube, with nascent trabeculae developing under the cardiomyocytes. We therefore envisage that our spatially patterned model could be used to study aspects of early human cardiac development, which is currently restricted to *in vivo* animal experiments. These cardiac microchambers can also be used for disease modelling during development with hiPSCs derived from patients with congenital cardiac diseases. However, we suggest that the model is limited to the earlier stages of heart development, before the start of looping, since the microchambers are artificially attached to the solid substrate.

Understanding the mechanisms of tissue formation is particularly important in cardiac development, since cardiac defects are the most commonly reported birth defects. This study offers a robust method to create patterned colonies of hiPSCs and generate highly defined cardiac microchambers. The biophysical cues of each pattern promoted spatially distinct cell density via cell condensation, with cells adjacent to the perimeter expressing an OCT4+ annulus coincident with the region of higher cell density, distorted cell shape, higher cell proliferation and E-cadherin expression. These early, spatially distinct differentiation processes were manifested in each pattern by integrating biochemical and biophysical cues, which directed the ultimate cardiac differentiation and microchamber structure, such that cells in the centre differentiated into cardiomyocytes while cells on the perimeter differentiated into myofibroblasts. This material-based differentiation approach is an important tool that can be used to manipulate biomechanics to induce changes in signal transduction and downstream cellular processes that are required for tissue morphogenesis. This study also shows that we can develop advanced *in vitro* models that link the biochemical and biophysical events of early cardiac development. Future studies on lineage tracking, and morphogen gradient and fate mapping, will further elucidate linkages between early cardiac development and microchamber formation. Accordingly, these well-defined hiPSC-based cardiac microchambers can be used to study the emergent properties of early heart development, in both healthy and diseased patients, which will be particularly important in the study of birth defects related to cardiac development. Furthermore, we envision these microchambers to be useful for drug-screening technologies (for example, developmental toxicity during pregnancy), tissue-based ‘organs on a chip’ or as natural pumping system in microfluidic systems.

## Methods

### PEG-patterned substrate

SU-8 masters with different features were fabricated using standard photolithography method with SU-8 50 (Microchem Co.), treated by oxygen plasma at 200 W for 1 min and salinized overnight with (heptadecafluoro-1,1,2,2 -tetrahydrodecyl) trimethoxysilane. The next day, to make a PDMS mask with clear-through holes, PDMS was mixed, degassed and a small amount was dropped on the SU-8 master. A plastic transparency was placed on top of the PDMS, and then the SU-8 master covered by the PDMS and the transparency was sandwiched by two glass slides and clamped by two paper clamps. The clamped master construct was placed in a 60 °C oven and cured overnight. The PDMS mask was carefully removed from the SU-8 master, sterilized with 70% ethanol and dried completely with a nitrogen gun.

To graft a thin layer of PEG on the commercial standard six-well TCPS plates, the plates were first treated with oxygen plasma at 425 W for 5 min. An 800-μl volume of a PEG mixture (15 ml PEG1000, 1.8 ml PEGDA, 14.55 ml IPA, 0.45 ml MilliQ water and 15 ml Irgacure 2959) was added into each well and the surface was evenly covered. The PEG was photo-crosslinked with ultraviolet exposure for 45 s using ultraviolet curing flood lamps (2000-EC Modular, Dymax Co.) and washed with MilliQ water three times to remove the uncrosslinked monomers. The PEG-grafted TCPS was sprayed with 70% ethanol, covered by PDMS masks and placed in 60 °C oven for 30 min. Finally, the whole plate was treated with two cycles of 3-min oxygen plasma at 200 W with a 3-min break in between^34^. The plates were transferred into a tissue culture lamina hood, exposed to 70% ethanol for 30 min, then washed with DPBS three times and coated with Matrigel (BD Bioscience hESC-qualified) for 30 min before the hiPSC seeding. To coat the patterned surfaces with ECM molecules, the plates were incubated with either fibronectin (50 μg ml^−1^) or vitronectin (50 μg ml^−1^) for 30 min at 37 °C. The chemicals used to create this PEG-patterned substrate are listed in Supplementary Table 2.

### Surface chemistry characterization

The XPS spectra were taken on a Surface Science Instruments S-probe spectrometer. X-ray spot size for these acquisitions was ∼800 m. Pass energy for survey spectra was 150 eV and pass energy for high-resolution scans was 50 eV. The Service Physics Hawk Data Analysis Software was used to determine peak areas, to calculate the elemental compositions from peak areas above a linear background and to peak fit the high-resolution spectra. The binding energy scales of the high-resolution spectra were calibrated by assigning the lowest binding energy peak a binding energy of 285.0 eV.

ToF-SIMS images were acquired on a ToF-SIMS 5 spectrometer (IonToF) using a 25 keV Bi_3_+ cluster ion source in the pulsed mode. The ion source was operated with a current of ∼0.07 pA at the surface. Secondary ions of a given polarity were extracted and detected using a reflectron time-of-flight mass analyser. Positive ion images were calibrated using the CH3^+^, C2H3^+^, C3H5^+^ and C7H7^+^peaks. The negative ion images were calibrated using the CH^−^, OH^−^, C2H^−^ and C4H^−^ peaks. PEG-related peaks were characterized as C2H5O, C3H3O, C3H7O, C4H7O2 and C4H9O2, whereas TCPS-related peaks were characterized as C4H9, C7H7, C8H9 and C9H7.

### Patterning of H9 hESCs and WTC hiPSCs

H9 hESCs, WTC hiPSCs, WTC H2B-mCherry hiPSCs were a gift from Bruce R. Conklin (Gladstone Institute). They were maintained in mTeSR1 media. Three days before the CHIR treatment (Day 0), hiPSCs were dissociated with Accutase (Invitrogen) for 5 min at 37 °C and seeded with 1 ml cell suspension in mTeSR1 containing 10 μM Y-27632 (Stemgent) on Matrigel-coated PEG-patterned substrate. The cell density was optimized for 200-μm diameter circle patterns with 0.4 million cells per ml, 400 μm with 0.8 million cells per ml and 1.2 million cells per ml. The cell-seeding density was optimized for the cells taking the same time (3 days) to reach confluence on the different sized patterns, so that we would have a uniform cell/surface condition to start the CHIR treatment. At 24 h after seeding, the PDMS masks were carefully removed from the PEG-patterned substrate using sharp tweezers, and the medium was changed to mTeSR1 without Y-27632 dislodging unattached cells. It took ∼3 days for hiPSCs (Day 0 to Day 3) to proliferate to fill up patterns.

### Generation of 3D cardiac microchamber

On Day 3, the patterned hiPSCs were treated with 12 μM of a GSK3 inhibitor (CHIR9902; Selleckchem) in RPMI 1640 medium containing B27 supplement without insulin (RPMI/B27-I; Life Technologies) for 24 h. The medium was changed on Day 4 to RPMI/B27-I and incubated for 48 h, followed by a 48-h treatment with 5 μM of an inhibitor of Wnt production (IWP-4, Stemgent) beginning on Day 6. On Day 8, the medium was changed to RPMI/B27-I for 2 days, and then changed to RPMI 1640 containing B27 complete supplement (RPMI/B27-C) on Day 10. The formation of 3D microchambers started from Day 4 after CHIR treatment, and the cardiac microchambers started beating at around Day 15.

### Immunocytochemistry and image analysis

The patterned cells were fixed with 4% (vol vol^−1^) paraformaldehyde for 15 min, permeabilized with 0.2% Triton X-100 for 5 min and blocked with 2% BSA, 4% goat serum and 0.1% Triton X-100 for 30 min. The samples were then incubated with primary antibodies for 2 h and secondary antibodies for 1.5 h. 4,6-diamidino-2-phenylindole (DAPI) was used to stain cell nuclei. For bright-field and epifluorescent microscopy, the images were taken using a Nikon Eclipse TS100F microscope with SPOT Flex camera. For confocal microscopy, the images were taken with a Zeiss LSM710 laser-scanning microscope in the Biological Imaging Facility at UC Berkeley. For two-photon microscopy, the images were taken with Prairie Ultima two-photon microscope in Berkeley CIRM/QB3 Shared Stem Cell Facility. On the basis of the Z-stack confocal images, the 3D cardiac microchambers were reconstructed in ImageJ. The antibodies and their dilutions used in this study are listed in Supplementary Table 3.

Individual cells were located and counted using ImageJ with Image-based Tool for Counting Nuclei (ITCN) package (www.bioimage.ucsb.edu/itcn.html). The percentage of OCT4+ cells in each pattern was calculated on the basis of the nuclei counting in DAPI staining image and OCT4 staining image. The MATLAB (Mathworks, Natick, MA) code used to create the cell density heatmap dissected the circle patterns into a series of 15-μm-thick annuli and assigned the cells to an annulus based on their location. To correlate the relationships among OCT4, E-cadherin and cell colony thickness on the asymmetric pattern, lines were constructed as vectors orthogonal to a polygonal approximation of the inner geometry as shown in Supplementary Fig. 5b. Owing to the limited field-of-view of high-magnification confocal microscope, a total of four images were taken and stitched in ImageJ. The average of fluorescent intensities of OCT4 and E-cadherin for each thickness were normalized by fluorescent intensity of DAPI and plotted according to thickness (Supplementary Fig. 5c,d). These measurements were fit to a two-term power function (*y=ax*^*b*^*+c*) using the MATLAB Curve Fitting Toolbox to calculate the coefficients with 95% confidence and fitting parameters including SSE, RMSE and *R*^2^. The nucleus shape index was calculated by dividing the longest axis of nuclei by the shortest axis (Supplementary Fig. 8b). Spatial cell density ratio was calculated by dividing the cell density at the perimeter by the centre.

### EdU incorporation study

Assessing the cell proliferation is normally achieved by directly measuring DNA synthesis, which initially is performed by antibody-based detection of the nucleoside analogue bromodeoxyuridine. The EdU (Click-iT EdU Alexa Fluor 594 Imaging Kit, Life Technologies) assay is a novel alternative to the bromodeoxyuridine assay. EdU is a nucleoside analogue of thymidine and is incorporated into DNA during active DNA synthesis. We used this assay to study the cell proliferation on the patterns during the CHIR treatment. After 16-h CHIR treatment, we replaced half of the media with fresh media containing EdU to reach the final EdU concentration of 20 μM and incubated it for 2 h. Then, the cells were consecutively fixed, permeablized, EdU-detected with Click-iT reaction cocktail and finally stained the nuclei with DAPI. Images were taken using a Nikon Eclipse TS100F microscope with a SPOT Flex camera. The percentage of EdU+ cells in each pattern was calculated on the basis of the nuclei counting in DAPI- and EdU-staining images.

### RT–qPCR analysis

The gene expression level for the patterned cells was analysed using RT–qPCR on Day 4 after the CHIR treatment. Adherent cells were washed with DPBS and homogenized with 1 ml TRIzol LS reagent (Invitrogen). Total RNA was collected and purified using the RNeasy Mini Kit (Qiagen). The total RNA concentration was quantified using a Nanodrop spectrophotometer and integrity was determined using the Agilent BioAnalyzer. Conversion of total RNA to cDNA was carried out using SuperScript III Reverse Transcriptase (Invitrogen) with random primers. qPCR was performed on the Applied Biosystems StepOnePlus instrument with customized target arrays in 96-well format (SA Biosciences/Qiagen), using SYBR Green ROX MasterMix (Qiagen). The data were analysed using 2^−Δ*C*t^ method relative to level of the housekeeping gene. To compare the pattern size effect on the gene expression before and after the CHIR treatment, the gene expression level of cells on the 200- and 600-μm patterns was normalized by gene expression of cells in the 400-μm patterns and plotted as a heatmap. The normalized gene expression >1 means that the gene was upregulated compared with 400-μm patterns, otherwise the gene was downregulated compared with 400-μm patterns. To study the early stage of differentiation by activation of the WNT pathway, the gene expression level of cells on 600-μm patterns after the CHIR treatment was normalized by cells before CHIR treatment and plotted as log_10_ value. The normalized gene expression >0 means that the gene was upregulated after CHIR treatment; otherwise, the gene was downregulated. Each experiment was carried out in four independent biological replicates. The genes used in this study are listed in Supplementary Table 4.

### Single-cell migration tracking

On Day 3, WTC hiPSCs with nuclear-localized mCherry (H2B-mCherry)^35^ patterned as 400-μm circles were treated by CHIR and the plate was transferred into the ImageXpress Micro XLS System (Molecular Devices), which was able to maintain 37 °C and 5% CO_2_. Both bright-field and epifluorescent images on multiple patterns within one well were taken every 30 min for 24 h. Owing to the high-speed motion of microscope stage to locate different patterns, the soluble chemical gradient within one pattern would be disrupted during the time-lapse microscopy. The consecutive 49 time-lapse images were reconstructed using the MetaXpress Software as TIFF stacks for single-cell migration analysis.

Cell movement was tracked with the custom-made MATLAB (Mathworks) code with image processing. For each pattern, 10 cells were randomly picked with five in the centre and five close to the perimeter, and totally five patterns were used for tracking analysis. The cell position data were recorded as a series of (*x, y*) coordinates with time for each cell. From each image, identified cells were tracked and matched, and relative cell displacements (d*x* and d*y*) between the matched mCherry-labelled nucleus centres of cells were measured for each time step. Line traces were plotted to provide the path of cell migration by connecting the positions of single cells in time sequence during migration. The initial position of each cell was assigned in the coordination of the pattern. If the radial distance between the initial position of the tracked cells and the pattern centre was larger than 100 μm, the cells were classified as ‘cells at the perimeter’ with blue trace lines; otherwise, the cells were classified as ‘cells in the centre’ with green trace lines.

The persistence random walk model^36^ was used for characterizing the ‘biased’ or ‘random’ cell migration and motility. The mean square displacement 
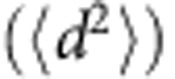
 with time increment as a function of time interval *t*=*k*Δ*t* was calculated using overlapping time interval sample method (equation (1)).




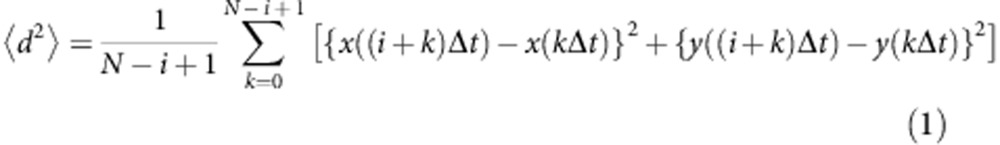




where *N* is the total number of time intervals, Δ*t* is interval of time and {*x*(*k*Δ*t*), *y*(*k*Δ*t*)} is spatial coordinate (Supplementary Fig. 6a).

By fitting the following theoretical expression (equation (2)) to the calculated mean squared displacement (equation (1)), we could obtain the persistent time (*P*) and random motility coefficient (*μ*) for 2D migration (*n*_*d*_*=2*).




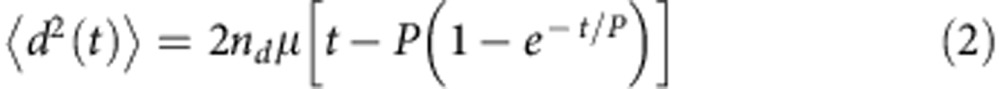




Persistent time (*P*) represents the average time length between drastic direction changes of cell migration. The *μ* indicates the quantitative measurement of random migration of cell population. For 2D migration, equation (2) can be derived as equation (3)




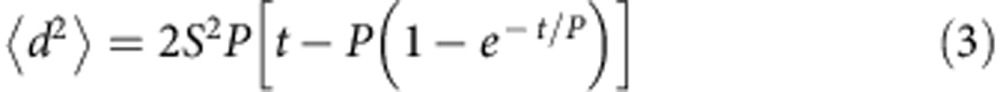




Where *S* is the cell migration velocity. On the basis of equations (2) and (3), the *μ* can be defined by equation (4) for 2D migration. The *P* and *μ* were calculated for up to one-third of the total migration duration (*t*_max_=*N*Δ*t*) to reduce the error.




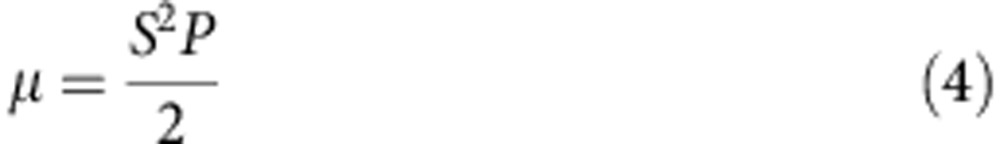




The cells whose experimental displacement data fit the theoretical persistence random walk model were considered as ‘biased’ cell migration, whereas the cells that did not fit the theoretical curve were considered as ‘random’ cell migration, as shown in Supplementary Fig. 6. To quantify the percentage of ‘biased’ cell migration in the centre or on the perimeter, we set a threshold on *μ* higher than 5 (about three times higher than the average value of *μ* for the cell migration at the perimeter) considered as ‘biased’ cell migration.

### Cardiomyocyte motion-tracking analysis

On Day 20, we recorded the cardiomyocytes beating at 20 frames per second for overall 500 frames using a Nikon Eclipse TS100F microscope with temperature-controlled stage and QImaging Retiga 4000R digital camera. Videos of beating 3D cardiac microchambers were exported as a series of single-frame image files and analysed using in-house developed motion-tracking software based on MATLAB (Mathworks). The software can automatically output the contraction heatmap and motion waveform for calculation of beat frequency and maximal contraction velocity^37^. The software is available at http://gladstone.ucsf.edu/46749d811/.

### Drug toxicity study

To assess the effect of Thalidomide on the formation of cardiac microchambers, we differentiated control groups using the normal protocol, and for drug groups 80 μg ml^−1^ Thalidomide was added starting from the CHIR treatment and continuing until the day that the cardiac microchambers were prepared for immunostaining (Day 20). This experimental design mimicked continuous drug exposure during pregnancy. The morphology of cardiac microchambers was assessed using confocal microscopy and reconstruction of the 3D images. The contraction behaviour of the cardiac microchambers was assessed by video recording and motion-tracking software. The changes in height, half width half maximum, beat frequency and contraction velocities of the cardiac microchambers with drug exposure were compared with the control groups with no drug administration.

### Statistical analysis

Data were presented as mean±s.d. For single comparisons, a two-sided Student’s *t*-test was used. For multiple comparisons, one-way analysis of variance was used with *post hoc* Tukey tests. *P*<0.05 was considered significant.

## Additional information

**How to cite this article:** Ma, Z. *et al*. Self-organizing human cardiac microchambers mediated by geometric confinement. *Nat. Commun.* 6:7413 doi: 10.1038/ncomms8413 (2015).

## Supplementary Material

Supplementary Information Supplementary Figures 1-12 and Supplementary Tables 1-4

Supplementary Movie 1 hESCs-derived cardiac tissue from a 39 day old embryoid body

Supplementary Movie 2 24-h time-lapse video captures cell migration within a 400-μm pattern during the EMT

Supplementary Movie 3 Reconstructed confocal microscopy image to visualize a 3D cardiac microchamber generated from WTC hiPSCs

Supplementary Movie 4 Reconstructed two-photon microscopy image to visualize the inside structure of a 3D cardiac microchamber

Supplementary Movie 5 Motion-tracking analysis on beating video of a cardiac microchamber generated from H9 hESCs

Supplementary Movie 6 Motion-tracking analysis on beating video of a cardiac microchamber generated from WTC hiPSCs
