# Supplementary material for: Self-organizing human cardiac microchambers mediated by geometric confinement
Source: Nat Commun. 2015 Jul 14;6:7413. doi: 10.1038/ncomms8413 (PMC4503387; doi:10.1038/ncomms8413)
Supplement: Supplementary Information — Supplementary Figures 1-12 and Supplementary Tables 1-4 [file ncomms8413-s1.pdf]

**Supplementary Figures**

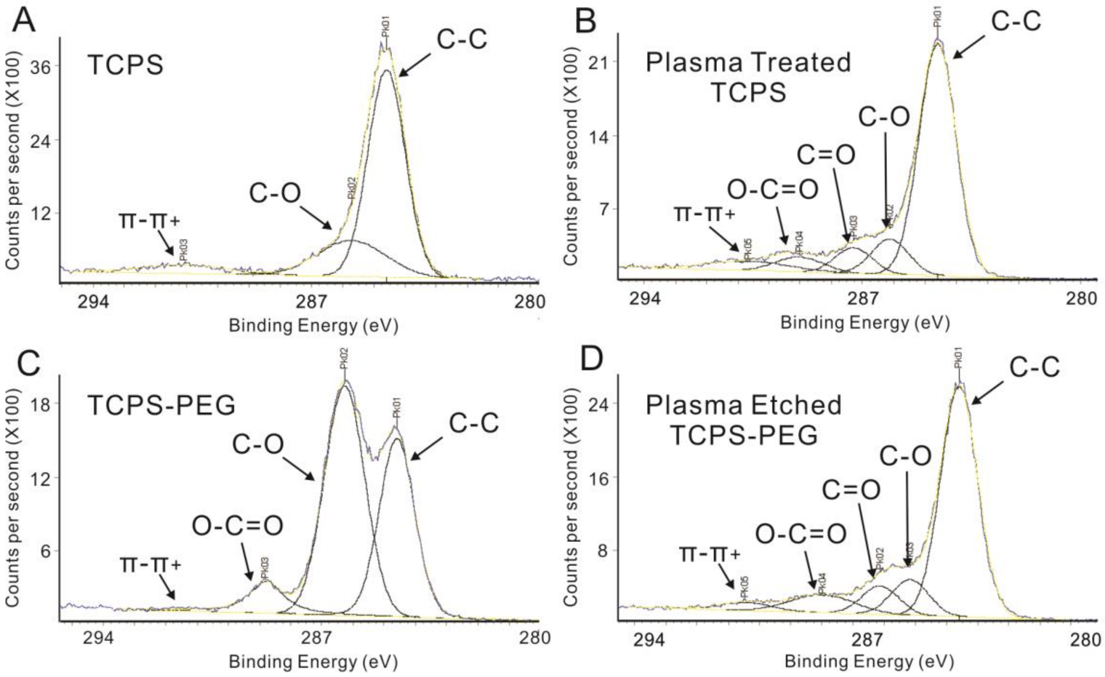


**Supplementary Figure 1. Surface chemistry characterization using XPS.** The spectra of (A) untreated TCPS, (B) oxygen plasma-treated TCPS, (C) PEG-grafted on the TCPS, and (D) oxygen plasma-etched PEG from the TCPS.

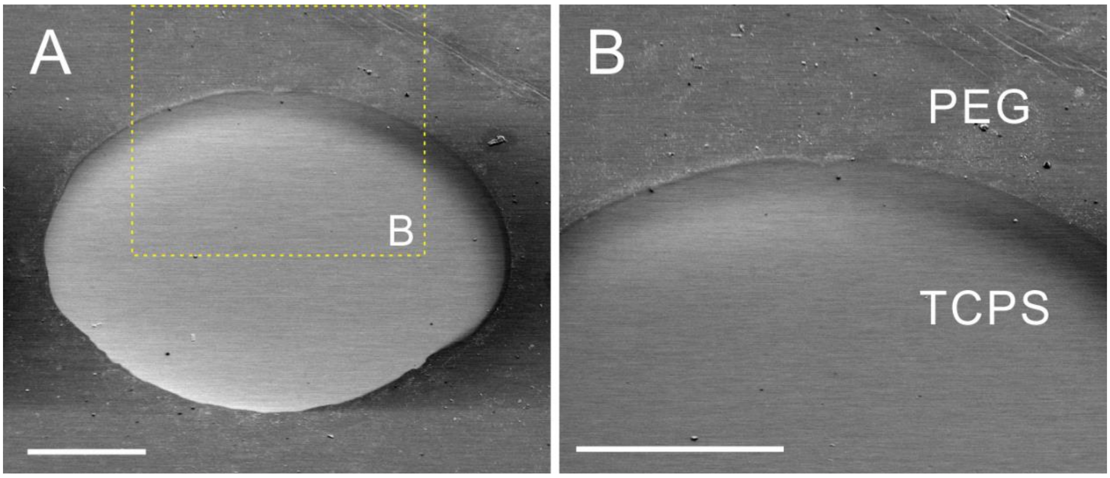


**Supplementary Figure 2. Surface morphology of etched area.** SEM image of a 600-µm pattern showing a thin layer of PEG outside the etched circle pattern. All scale bars 100 µm.

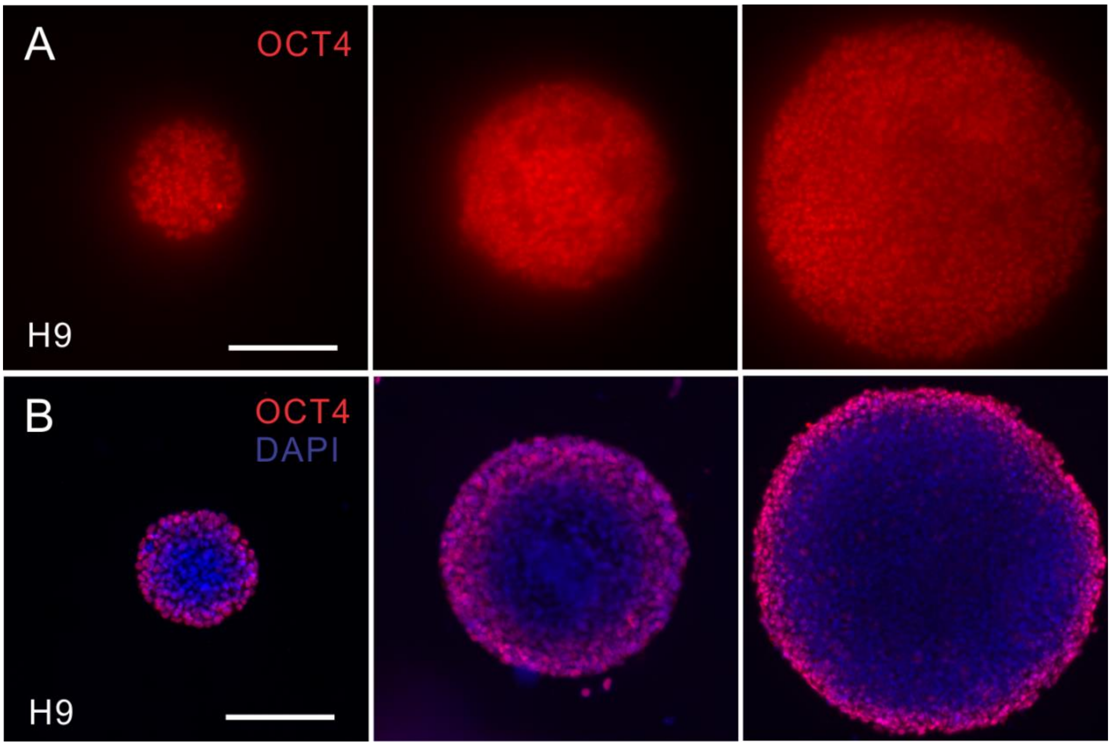


**Supplementary Figure 3. Patterning and spatial differentiation of hESCs.** PEG-patterned circles with different diameters (200 µm, 400 µm and 600 µm) created organized colonies of H9 hESCs. The cells showed (A) uniform OCT4 expression before the CHIR treatment, and (B) a pronounced OCT4+ annulus after CHIR treatment. All scale bars 200 µm.

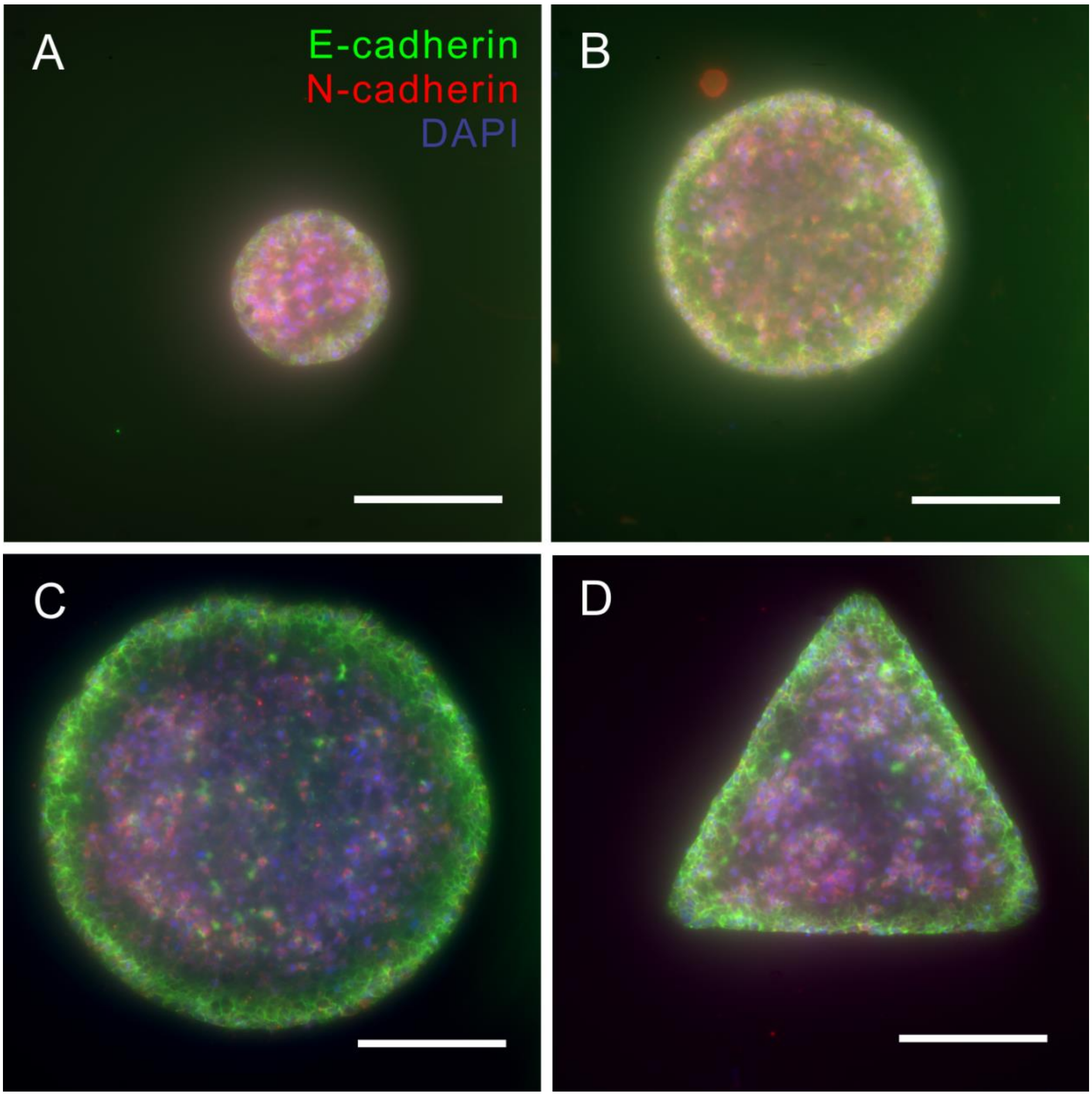


**Supplementary Figure 4. Spatial epithelial-mesenchymal transition (EMT) within the patterns.** Treated by CHIR to induce the EMT, the cells at the pattern perimeter expressed high levels of E-cadherin, indicating an epithelial phenotype, whereas cells in the center expressed high levels of N-cadherin, indicating a mesenchymal phenotype. This spatial differentiation and epithelial-mesenchymal interface was observed on all the patterns (A) circle with 200 µm diameter, (B) circle with 400 µm diameter, (C) circle with 600 µm diameter, and (D) triangle with 540 µm leg length. All scale bars 200 µm.

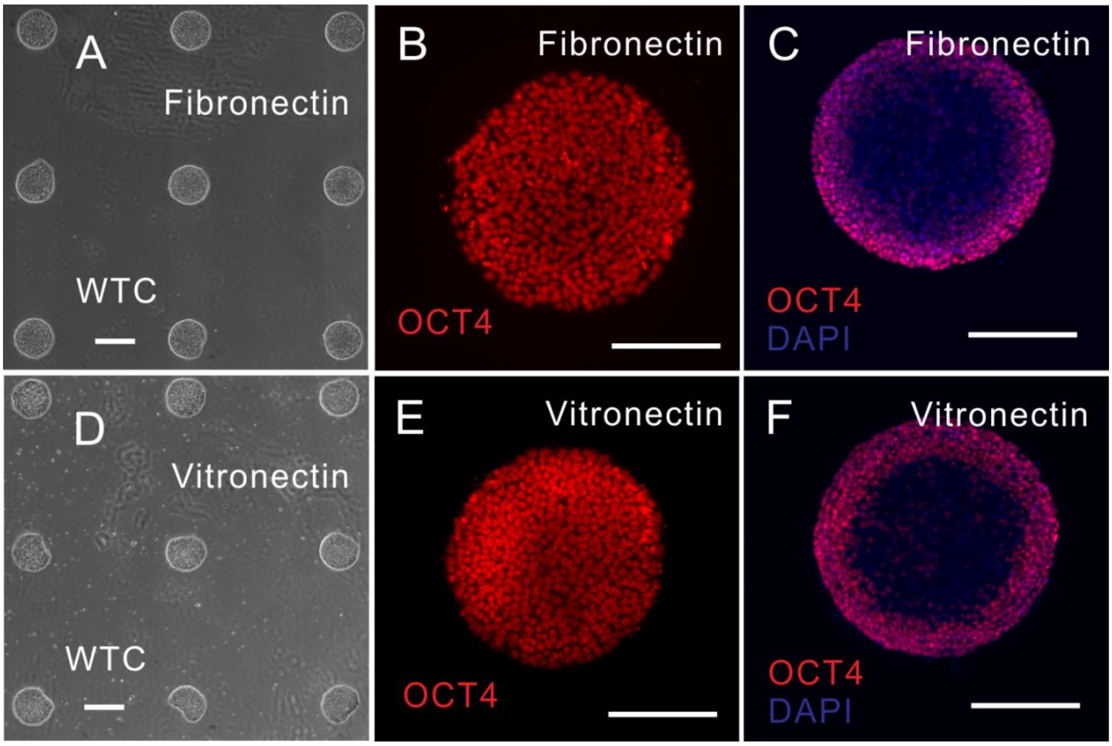


**Supplementary Figure 5. Patterning and spatial differentiation of hiPSCs on ECM protein coated surfaces.** hiPSCs were patterned with the PEG-based method and then coated with (A) fibronectin and (D) vitronectin. Scale bars 400 µm. The cells showed uniform OCT4 expression before the CHIR treatment (B & E), and a pronounced OCT4+ annulus after CHIR treatment (C & F). Scale bars 200 µm.

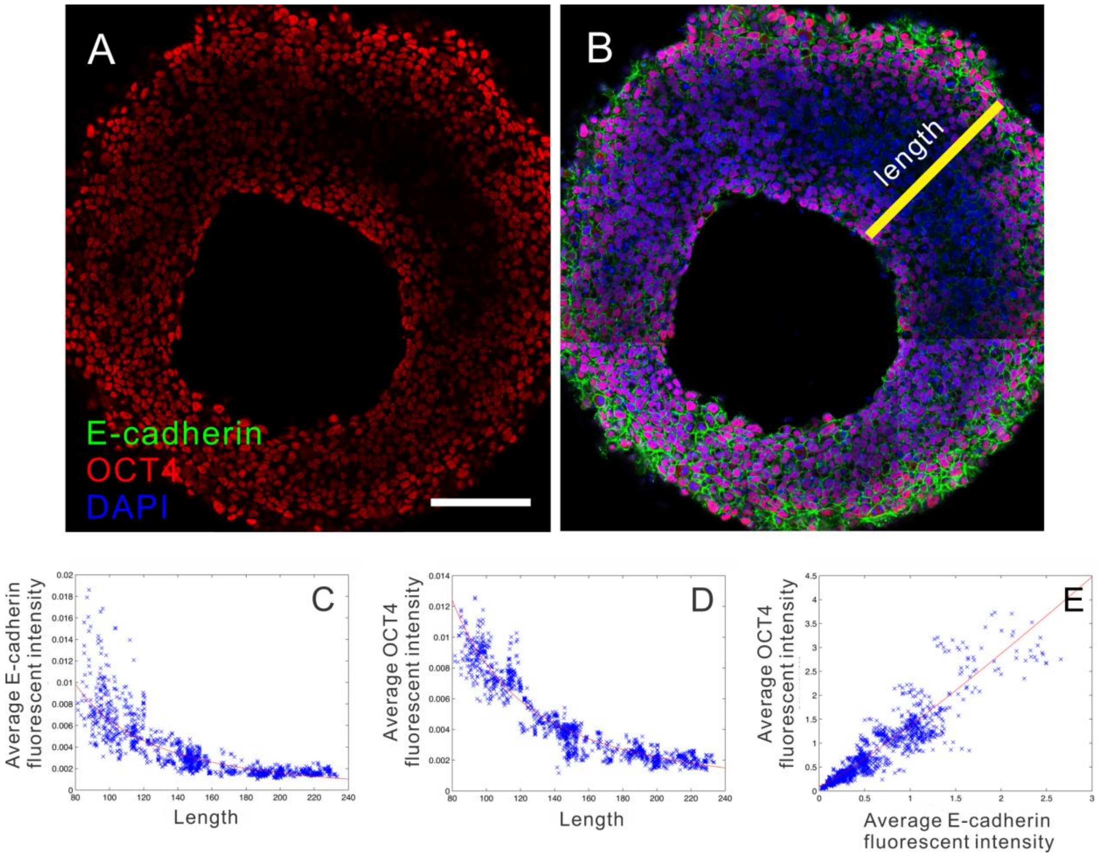


**Supplementary Figure 6. An asymmetric donut-shape pattern was created to vary the local mechanical stress.** Narrow areas had (A) more OCT4+ cells with (B) higher cell density and higher E-cadherin expression. Averaging the E-cadherin and OCT4 fluorescent intensity by the length of the line trace across the pattern (represented as the yellow line in B, the smallest length was around 80 µm and largest one was around 240 µm), the narrower region of the pattern had higher (C) E-cadherin ($R^2$ = 0.6484) and (D) OCT4 expression ($R^2$ = 0.8533), and (E) the expression of E-cadherin and OCT4 was highly correlated ($R^2$ = 0.8250). Scale bar 100 µm.

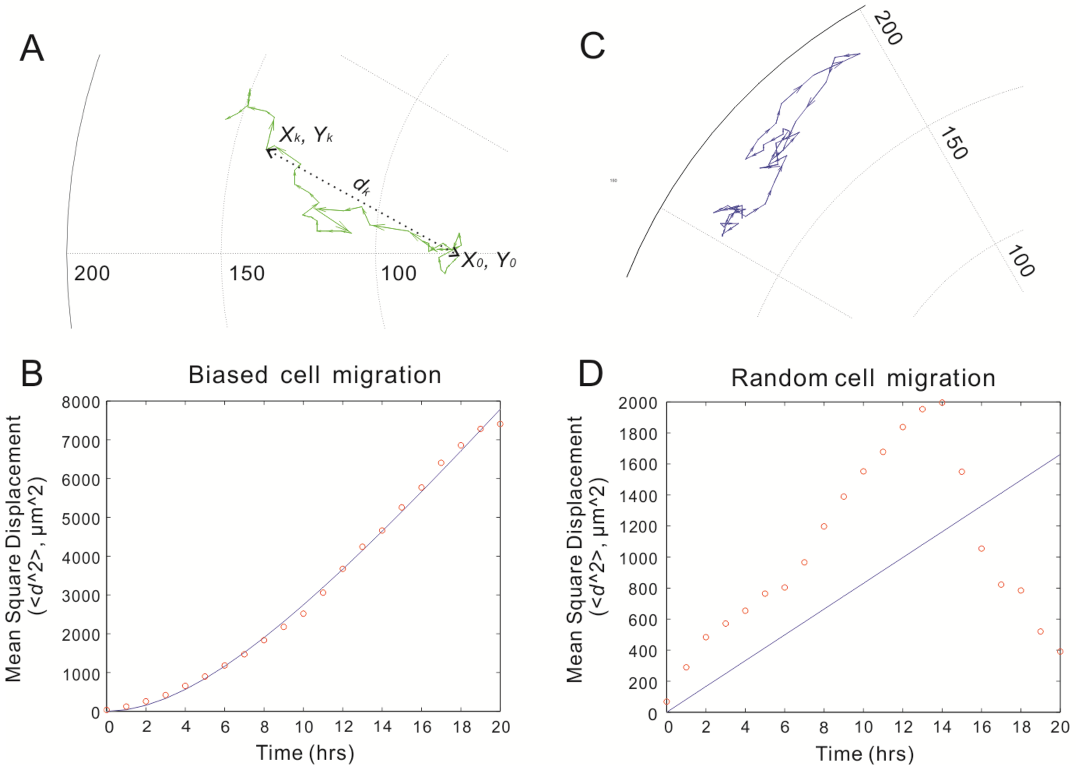


**Supplenmentary Figure 7. Single-cell migration tracking and analysis based on persistence random walk model.** Examples of (A) "biased" cell migration with (B) good curve-fitting of theoretical expression (equation (2) in Methods: Single-cell migration tracking) to the calculated mean squared displacement (equation (1) in Methods: Single-cell migration tracking) for motility coefficient and persistent time, and (C) "random" cell migration at the perimeter (D) that was poorly fit.

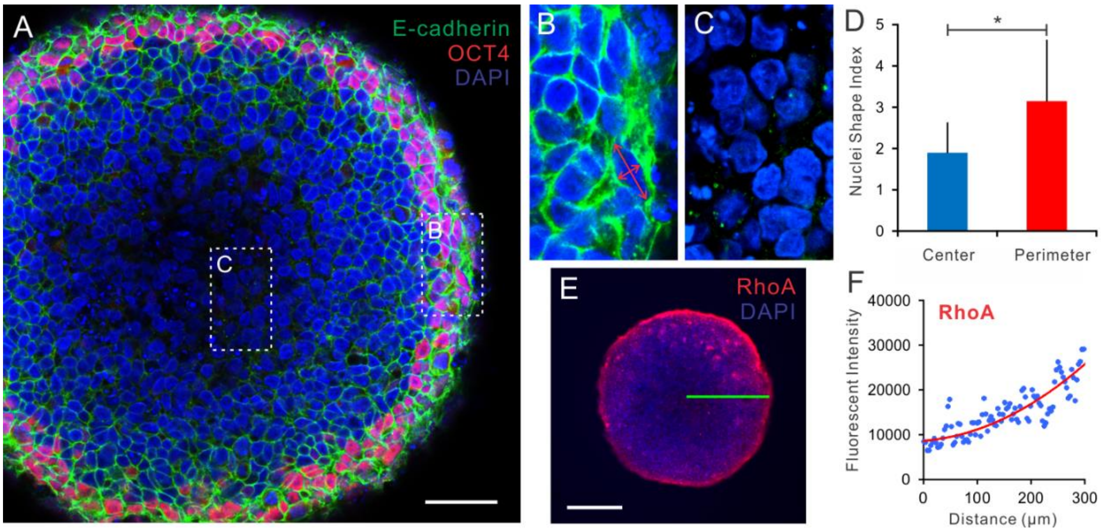


**Supplementary Figure 8. Cell elongation along the pattern perimeter.** (A) Confocal image of a 400-µm pattern clearly demonstrating an OCT4 annulus adjacent to the pattern perimeter. Scale bar 50 µm. (B) The elongated nuclei morphology of the cells was observed within the annulus, whereas (C) nuclei of cells in the center were not elongated shape. (D) Cells at the perimeter had a higher nuclei shape index compared to the cells in the center Data represent as the means with error bars S.D. with n = 20 individual patterns. Statistical comparison was made between center and perimeter using two-sided Student's t-test. $*p < 0.05$. (E) Epifluorescent image of a 600-µm pattern with a RhoA staining revealing increasing RhoA intensity towards the perimeter of the pattern as highlighted by (F) a radial cross-section of the fluorescence intensity ($R^2$=0.6975). Scale bar 200 µm.

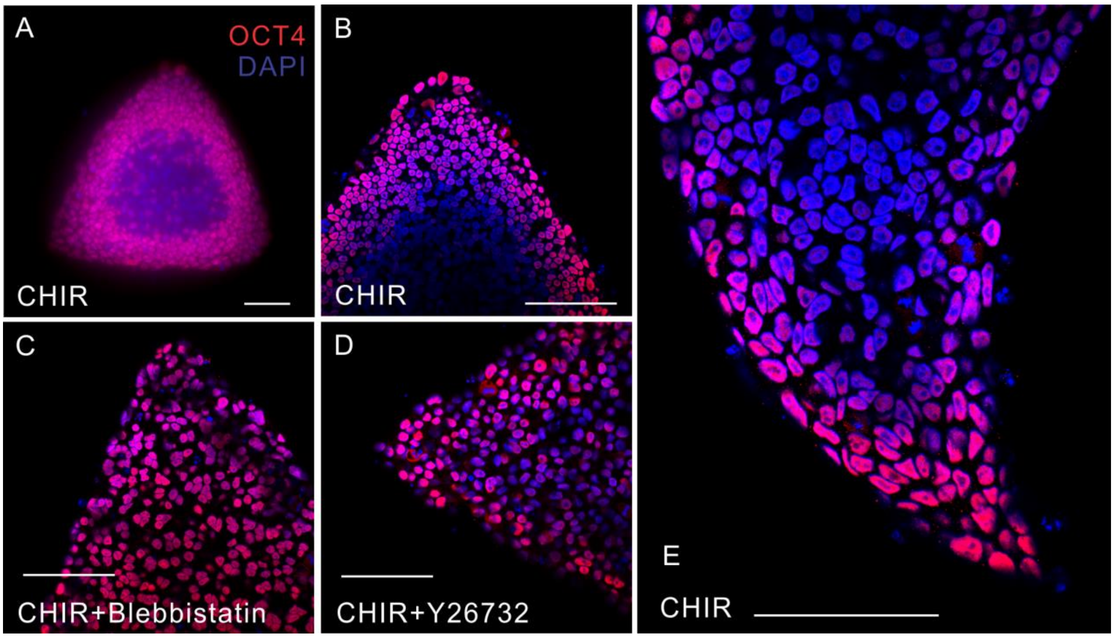


**Supplementary Figure 9. Spatial differentiation on triangle and shape-angled patterns.** We created (A & B) triangle patterns (540 µm in leg length), which promoted a perimeter pattern of OCT4+ cells similar to what we observed for circular patterns after CHIR treatment. The OCT4+ band was eliminated by either applying (C) Blebbistatin or (D) Y26732. (E) Shape-angled patterns increase the mechanical stress at the tip, causing cells to have highly elongated morphology. All scale bars 100 µm.

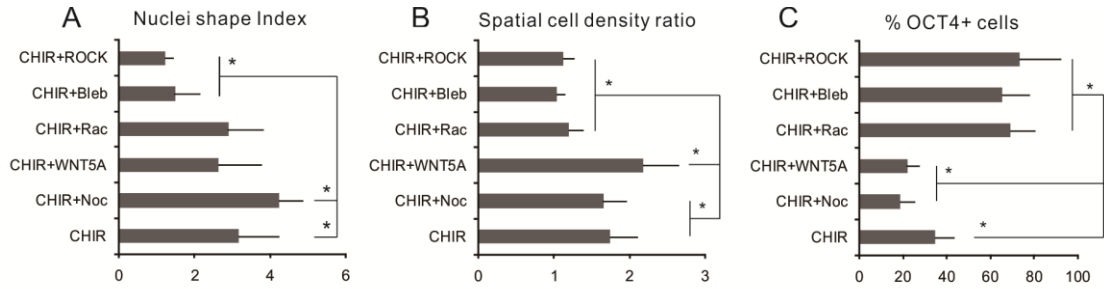


**Supplementary Figure 10. Effect of mechanotransduction modulators on cell condensation and OCT4+ expression.** The level of cell condensation was manifested as (A) nuclei shape index and (B) spatial cell density ratio. Mechanotransduction modulators significantly affected cell condensation, and (C) the percentage of OCT4+ cells on the entire pattern. Data represent as the means with error bars S.D. with n = 20 individual patterns for each experimental condition. Statistical comparison was made among different experimental conditions using one-way ANOVA with *post-hoc* Tukey tests. *$p < 0.05$.

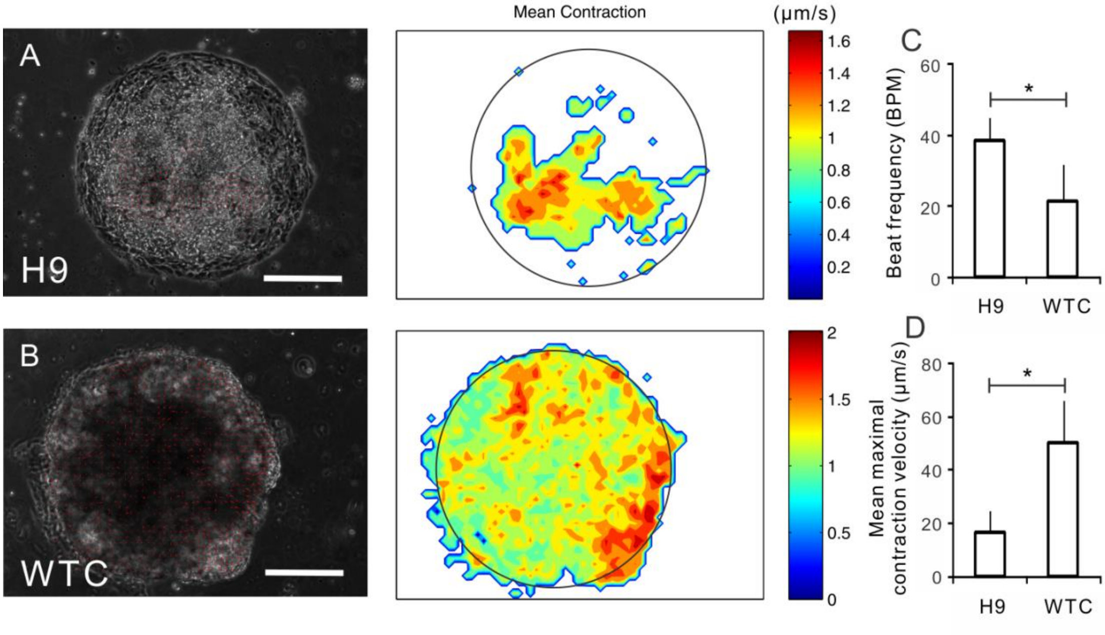


**Supplementary Figure 11. Contraction heat maps for 3D cardiac microchambers.** Using in-house developed motion-tracking software, we quantified the beating behavior of the 3D cardiac microchamber and created the contraction heat maps for the microchambers generated from (A) H9 hESCs and (B) WTC hiPSCs. The cardiac microchambers generated from WTC hiPSCs had (C) lower beat frequency, and (D) the higher contraction velocity compared to microchambers generated from H9 hESCs. Data represent as the means with error bars S.D. with n = 6 for each cell line. Statistical comparison was made between H9 hES-derived microchambers and WTC hiPS-derived microchambers using two-sided Student's t-test. *$p < 0.05$. All scale bars 200 μm.

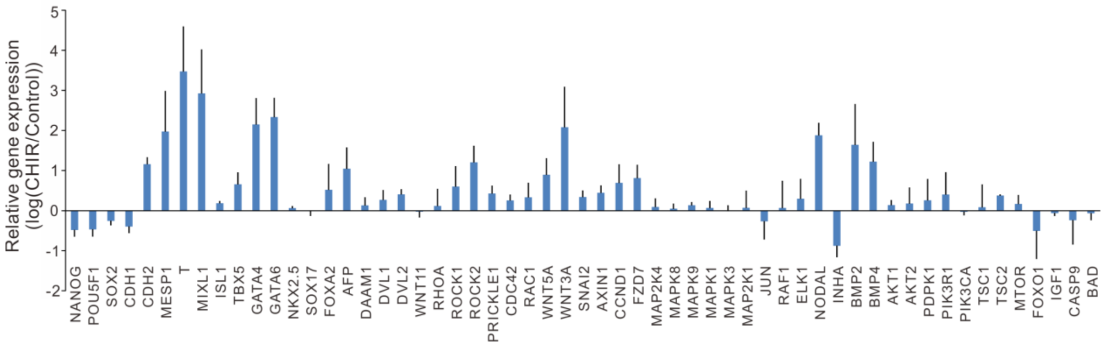


**Supplementary Figure 12. Gene expression on early stage of hiPSC differentiation.** hiPSCs patterned as 600-μm circles treated by CHIR exhibited reduction in pluripotent markers, EMT induction, mesoderm specification, expression of NODAL, BMP, and WNT signaling and onset of cardiac differentiation with increases in cardiac progenitor markers. Data represent as the means with error bars S.D. with n = 4 independent biological replicates.

## Supplementary Tables

**Supplementary Table 1. Modulators of mechanotransduction with concentration used in this study**

| Compounds | Effects | Concentration | Catalog No. | Company |
|---|---|---|---|---|
| Y27632 | RhoA/ROCK inhibitor | 10 µM | 04-0012-02 | Stemgent |
| Blebbistatin | Myosin light chain kinase inhibitor | 10 µM | 203390-5MG | Calbiochem |
| NSC23766 | Rac1 inhibitor | 10 µM | 553502-5MG | Calbiochem |
| Nocodazole | Microtubule interferer | 20 nM | 487929-10MG | Calbiochem |
| WNT5A recombinant protein | Enhancer of non-canonical WNT pathway | 1µg/mL | 645-WN-010/CF | R&D system |

**Supplementary Table 2. Chemicals used for producing PEG-patterned substrate**

| Chemicals | Catalog No. | Company |
|---|---|---|
| PEG1000 | 16666-100 | Polysciences |
| PEG diacrylate | 01871-250 | Polysciences |
| Irgacure 2959 | 410896 | Sigma-Aldrich |
| SU-8 50 | SU-8 50 | Microchem |
| Polydimethylsiloxane (PDMS) | Sylgard 184 silicone elastomer kit | Dow Corning |
| (heptadecafluoro-1,1,2,2-tetrahydrodecyl) trimethoxysilane | SIH5841.5 | Gelest |
| Isopropyl alcohol (IPA) | W292907 | Sigma-Aldrich |

**Supplementary Table 3. Antibodies used in this study**

| Antibodies | Species | Dilutions | Catalog No. | Company |
|---|---|---|---|---|
| OCT4 | Rabbit | 1:200 | ab19857 | Abcam |
| E-cadherin | Mouse | 1:50 | ab1416 | Abcam |
| N-cadherin | Rabbit | 1:100 | ab12221 | Abcam |
| NANOG | Rabbit | 1:200 | ab21624 | Abcam |
| SOX2 | Rabbit | 1:200 | ab92494 | Abcam |
| NKX2.5 | Rabbit | 1:50 | SC14033 | Santa Cruz Biotech |
| Cardiac Troponin T | Mouse | 1:200 | MS295P | Thermo Scientific |
| Sarcomeric α-actinin | Mouse | 1:300 | A7811 | Sigma Aldrich |
| Myosin heavy chain | Mouse | 1:200 | ab97715 | Abcam |
| SM22 | Rabbit | 1:300 | ab14106 | Abcam |
| Calponin | Rabbit | 1:200 | ab46794 | Abcam |
| Smooth muscle actin | Rabbit | 1:100 | ab5694 | Abcam |
| RhoA | Rabbit | 1:100 | ab68826 | Abcam |
| DAPI | - | 2 drops/mL | R37606 | Invitrogen |

**Supplementary Table 4. PCR Primer with accession number used in this study**

| | | | | | |
|---|---|---|---|---|---|
| AFP | AKT1 | AKT2 | AXIN1 | BAD | BMP2 |
| NM_001134 | NM_005163 | NM_001626 | NM_003502 | NM_032989 | NM_001200 |
| | | | | | |
| BMP4 | CASP9 | CCND1 | CDC42 | CDH1 | CDH2 |
| NM_001202 | NM_001229 | NM_053056 | NM_001791 | NM_004360 | NM_001792 |
| | | | | | |
| DAAM1 | DVL1 | DVL2 | ELK1 | FOXA2 | FOXO1 |
| NM_014992 | NM_004421 | NM_004422 | NM_005229 | NM_021784 | NM_002015 |
| | | | | | |
| FZD7 | GAPDH | GATA4 | GATA6 | HSP90AB1 | IGF1 |
| NM_003507 | NM_002046 | NM_002052 | NM_005257 | NM_007355 | NM_000618 |
| | | | | | |
| INHA | ISL1 | JUN | MAPK1 | MAPK3 | MAPK8 |
| NM_002191 | NM_002202 | NM_002228 | NM_002745 | NM_002746 | NM_002750 |
| | | | | | |
| MAPK9 | MAP2K1 | MAP2K4 | MESP1 | MIXL1 | MTOR |
| NM_002752 | NM_002755 | NM_003010 | NM_018670 | NM_031944 | NM_004958 |
| | | | | | |
| NANOG | NODAL | NKX2.5 | PDPK1 | PIK3CA | PIK3R1 |
| NM_024865 | NM_018055 | NM_004387 | NM_002613 | NM_006218 | NM_181523 |
| | | | | | |
| PRICKLE1 | POU5F1 | RAC1 | RAF1 | RHOA | ROCK1 |
| NM_153026 | NM_002701 | NM_006908 | NM_002880 | NM_001664 | NM_005406 |
| | | | | | |
| ROCK2 | SNAI2 | SOX2 | SOX17 | T | TBX5 |
| NM_004850 | NM_003068 | NM_003106 | NM_022454 | NM_003181 | NM_000192 |
| | | | | | |
| TSC1 | TSC2 | WNT3A | WNT5A | WNT11 | |
| NM_000368 | NM_000548 | NM_033131 | NM_003392 | NM_004626 | |
